# Antibacterial Activity of Equisetin from Endophytic *Fusarium incarnatum* JMB4 Associated with *Jatropha multifida* L. Against MRSA: Experimental and *In Silico* Insights

**DOI:** 10.1021/acsomega.6c07232

**Published:** 2026-10-06

**Authors:** Herland Satriawan, Rachel Shammara Khalda, Hao-Ze Li, Arif Setiawansyah, Ping-Chung Kuo, Dian Handayani

**Affiliations:** † School of Pharmacy, College of Medicine, 34912 National Cheng Kung University, 1 University Road, Tainan 70101, Taiwan; ‡ Laboratory of Sumatran Biota/Faculty of Pharmacy, Universitas Andalas, Kampus Limau Manis, Padang 25163, Indonesia; § Akademi Farmasi Cendikia Farma Husada, Jl. Pulau Enggano–Tirtayasa No. 100, Sukabumi, Bandar Lampung 35134, Indonesia

## Abstract

An antibacterial metabolite was isolated from the endophytic fungus *Fusarium incarnatum* JMB4 associated with *Jatropha multifida* L. by bioactivity-guided chromatography and recrystallization. The isolated compound was assigned as equisetin using UV–vis, FTIR, LC–MS/MS, and ^1^H NMR data. Anti-MRSA activity was evaluated by disk diffusion, with chloramphenicol and amoxicillin as antibiotic comparators. At 1% (w/v; 100 μg/disk), equisetin produced an inhibition zone of 23.94 ± 0.66 mm; the recorded comparator zones were 24.71 ± 0.68 mm for chloramphenicol (30 μg/disk) and 11.76 ± 0.66 mm for amoxicillin (25 μg/disk). The lowest tested equisetin concentration producing a measurable zone was 78.1 μg/mL (7.03 ± 0.25 mm). This diffusion end point does not represent a minimum inhibitory concentration (MIC), and the unequal disk loads preclude a direct comparison of intrinsic antibacterial potency. Exploratory docking against penicillin-binding protein 2a (PBP2a) yielded a score of −5.5041 kcal/mol and a predicted pose outside the canonical catalytic pocket. A 100 ns molecular dynamics simulation described the behavior of the modeled complex but did not establish PBP2a inhibition. These results document anti-MRSA activity of the isolated compound under the tested conditions; standardized MIC determination and further structural validation remain necessary.

## Graphical abstract

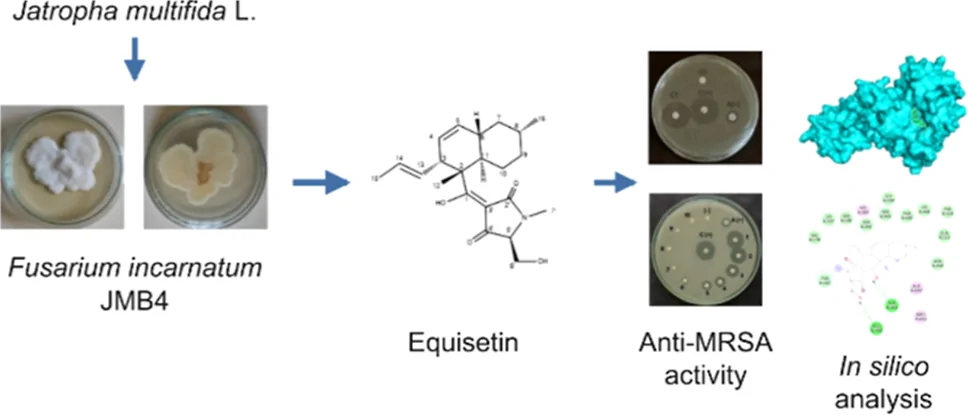

## Introduction

Methicillin-resistant *Staphylococcus aureus* (MRSA) remains one of the most consequential antimicrobial-resistant pathogens worldwide. The estimated number of deaths directly attributable to MRSA increased from approximately 57,200 in 1990 to 130,000 in 2021, representing the largest increase among the pathogen–drug resistance combinations evaluated globally.^1^ Consequently, the World Health Organization continues to classify MRSA as a high-priority pathogen for antibacterial research and development.^2^ Methicillin resistance is primarily associated with the mecA-encoded penicillin-binding protein 2a (PBP2a), which has a low affinity for most β-lactam antibiotics and enables cell–wall synthesis to continue in their presence.^3^ These challenges underscore the need to identify antibacterial compounds with alternative chemical scaffolds and potentially distinct modes of action.

Plant-associated endophytic fungi represent promising sources of antibacterial secondary metabolites. In particular, endophytic *Fusarium incarnatum* strains have demonstrated considerable biosynthetic capacity. For example, an endophytic *F. incarnatum* strain isolated from Cymbidium orchids produced several metabolites with antibacterial activity against clinically relevant bacteria.^4^ These findings indicate that the antibacterial potential and metabolite profiles of *F. incarnatum* may vary depending on the fungal strain and its host–plant association. *Jatropha multifida* L. is a medicinal plant traditionally used for treating wounds, skin infections, and inflammatory conditions, making its associated endophytic microorganisms relevant candidates for antibacterial bioprospecting.^5,6^

Previous screening of 14 endophytic fungal isolates from *J. multifida* identified *F. incarnatum* JMB4 as an isolate with anti-MRSA activity.^7^ The present work addresses the isolation and characterization of an antibacterial metabolite from this strain.

Previous experimental evidence identified bacterial acetyl-CoA carboxylase (ACC), particularly its biotin carboxylase component, as a probable molecular target of equisetin through inhibition of fatty acid biosynthesis.^8^ Nevertheless, because PBP2a is a central determinant of methicillin resistance in MRSA, the present study explored whether equisetin might additionally interact with PBP2a. This exploratory analysis was not intended to designate PBP2a as the sole or primary antibacterial target of equisetin.

This study aimed to isolate and characterize an antibacterial metabolite from *F. incarnatum* JMB4 associated with *J. multifida* and evaluate its anti-MRSA activity. Docking and molecular dynamics were used to explore a possible interaction with PBP2a.

## Materials and Methods

### General Experimental Procedures

All chemicals and reagents used in this study were of analytical grade. Thin-layer chromatography (TLC) was performed on precoated silica gel 60 F_254_ plates (0.2 mm, Merck, Darmstadt, Germany). TLC was used to monitor the fractions obtained from column chromatography, which were visualized under UV light at 254 and 366 nm with appropriate staining reagents. Preparative TLC was performed using benzene: acetone (10:1, v/v) as the mobile phase. UV–vis and FTIR analyses were conducted using standard spectrophotometers. Liquid chromatography–tandem mass spectrometry (LC–MS/MS) analysis was performed using an ultraperformance liquid chromatography system (ACQUITY UPLC Class-H System, Waters, USA) coupled to a Xevo G2-S quadrupole time-of-flight (QToF) mass spectrometer (Waters, USA). Chromatographic separation was achieved on an HSS C18 column (2.1 × 100 mm, 1.8 μm; Waters, USA) maintained at 50 °C, with the sample compartment maintained at 25 °C. The mobile phases consisted of water containing 5 mM ammonium formate as solvent A and acetonitrile containing 0.05% formic acid as solvent B. Gradient elution was performed as follows: 0–2 min, 95% A and 5% B; 2–3 min, 75% A and 25% B; 14–15 min, 100% B; and 19–23 min, 95% A and 5% B. The flow rate was 0.2 mL/min, the total run time was 23 min, and the injection volume was 5 μL. ^1^H NMR spectra were recorded on a 400 MHz spectrometer using CD_3_OD as the solvent.

### Fungal Material

The endophytic fungus *F. incarnatum* JMB4 was previously isolated from stem tissue of *J. multifida* L. by our research group.^7^ The plant was collected in Durian Taruang, Kuranji District, Padang City, West Sumatra, Indonesia (0°55′35.4″ S, 100°24′25.3″ E). The authenticated plant specimen was deposited at the ANDA Herbarium, Universitas Andalas, under voucher number RSM002.^7^ The fungal isolate was identified by rRNA gene sequencing, with the sequence reported under NCBI accession PZ176744. The strain is maintained at the Sumatra Biota Laboratory, Universitas Andalas. JMB4 was selected for fermentation and bioactivity-guided isolation on the basis of the previous antibacterial screening.

### Extraction and Isolation of the Compound

The fungus was cultivated on a solid rice-based medium (approximately 3.0 kg) under static conditions at room temperature for 4–6 weeks. The cultured medium was exhaustively extracted with ethyl acetate (EtOAc), and solvent fractionation yielded *n-hexane* (30.0 g), dichloromethane (DCM; 5.1 g), and methanol (1.5 g) fractions. Bioactivity-guided purification proceeded through the DCM fraction,^9^ which was separated by silica gel column chromatography using step-gradient and isocratic elution with *n-hexane*, DCM, EtOAc, and methanol. Fractions were monitored by TLC using toluene:DCM:EtOAc (6:3:1, v/v/v) with seven drops of formic acid. Fractions with similar TLC profiles were combined to yield 12 fractions. Fraction F5 (400 mg) was rechromatographed on silica gel with *n-hexane*:DCM (1:9, v/v), yielding nine subfractions (F5.1–F5.9). Subfraction F5.2 (10 mg) was further separated using toluene:DCM (1:3, v/v), yielding F5.2.1 and F5.2.2. Recrystallization of F5.2.1 afforded Compound **1** (4.6 mg). Analytical TLC showed a single spot in three solvent systems: EtOAc:DCM (4:1) containing formic acid, toluene:acetone (3:1), and toluene:DCM (1.5:3.5). LC–MS showed a dominant peak at 14.20 min. These observations supported chromatographic homogeneity but did not provide a quantitative purity measurement.

### Anti-MRSA Activity of the Isolated Compound

Nutrient agar (NA; 10 g in 500 mL distilled water) was used for the disk-diffusion assay and sterilized at 121 °C for 15 min^9^

MRSA was subcultured on NA at 37 °C for 18–24 h. The bacterial suspension was prepared in sterile physiological saline (0.9% NaCl), homogenized, and adjusted using a McFarland densitometer to 0.5 McFarland (approximately 1.5 × 10^8^ CFU/mL).^9^

A 100 μL aliquot of the standardized MRSA suspension was spread onto each NA plate. Compound **1** was dissolved in DMSO and tested at ten concentrations ranging from 1% to approximately 0.00195% (w/v). A 10 μL aliquot of each solution was applied to a sterile paper disk. Commercial chloramphenicol (30 μg/disk) and amoxicillin (25 μg/disk) disks served as antibiotic comparators, and DMSO served as the solvent control. After incubation at 37 °C for 24 h, inhibition-zone diameters were measured using a digital caliper.^10^ Assays were performed in triplicate, and results are reported as mean ± standard deviation (SD).

The diffusion end point was defined as the lowest tested solution concentration that produced a measurable inhibition zone. Concentrations were converted using 1% (w/v) = 10,000 μg/mL, and disk loads were calculated from the 10 μL application volume. Dilution-based MIC data are not available for Compound **1** or the antibiotic comparators in the reported data set. Accordingly, the diffusion end point is not reported as an MIC,^11^ and zone measurements are used only for descriptive comparison under the tested conditions.

### Molecular Docking Analysis

Molecular docking was performed using Molecular Operating Environment (MOE) version 2015.10 (Chemical Computing Group Inc., Montreal, Canada). The crystal structure of MRSA PBP2a in complex with piperacillin (PDB ID: 6H5O) was obtained from the RCSB Protein Data Bank.^3^ Equisetin was obtained from PubChem, and piperacillin was used as the reference ligand. PBP2a was examined as an exploratory target related to methicillin resistance. ACC was not included in the docking analysis; previous experimental evidence supporting ACC as an equisetin target^8^ is considered separately from the present PBP2a predictions.

Protein and ligand structures were prepared using the default MOE protocol, including hydrogen addition, protonation, and energy minimization with the MMFF94x force field. The active site was identified using the Site Finder module, with key residues including Ser403, Asn464, Tyr519, Gln521, Thr600, and Glu602. Docking was conducted using the MOE Dock module with Triangle Matcher placement and London dG scoring, followed by refinement and rescoring using GBVI/WSA dG. The best binding poses were analyzed for hydrogen bonding and hydrophobic interactions using Discovery Studio Visualizer 2024.

### Molecular Dynamics Simulation

Molecular dynamics simulations were conducted in GROMACS for 100 ns to examine the modeled protein–ligand complexes. The protein was parametrized with CHARMM36m and the ligand with CGenFF. Each complex was placed in an octahedral box containing TIP3P water and sodium/chloride ions, followed by energy minimization and equilibration at constant volume and then constant pressure. Temperature was maintained at 310 K using velocity rescaling, and pressure at 1 atm using the Parrinello–Rahman barostat. Long-range electrostatics were treated using the particle mesh Ewald method. Trajectories were analyzed for protein backbone root-mean-square deviation (RMSD), residue root-mean-square fluctuation (RMSF), radius of gyration (Rg), solvent-accessible surface area (SASA), and protein–ligand hydrogen bonds.^12,13^

## Results and Discussion

### Isolation and Purification of Metabolites

Bioactivity-guided chromatography and recrystallization afforded 4.6 mg of Compound **1** as a white powder. The purification route is described in Materials and methods; the following results focus on the isolated compound.

### Antibacterial Activity

In the disk-diffusion assay, the inhibition-zone diameter of Compound **1** decreased from 23.94 ± 0.66 mm at 1% (w/v; 100 μg/disk) to 7.03 ± 0.25 mm at 7.81 × 10^–3^% (approximately 78.1 μg/mL; 0.781 μg/disk). No measurable inhibition zone was recorded at the two lower tested concentrations (Figure 1; Table 1).

**Figure 1:**
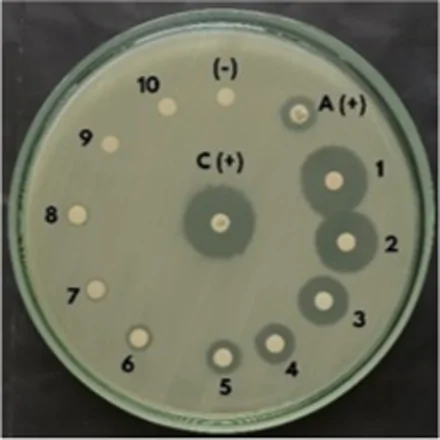
Disk-diffusion activity of Compound **1** (equisetin) against MRSA. Disk labels 1–10 correspond to the numbered equisetin rows in Table 1, with disk 1 representing the highest tested concentration (1% w/v; 100 μg/disk). A­(+), amoxicillin comparator (25 μg/disk); C­(+), chloramphenicol comparator (30 μg/disk); (–), DMSO control. The lowest tested equisetin concentration with a measurable zone was 78.1 μg/mL; this is a diffusion end point, not an MIC.

**Table 1:** Disk-Diffusion Activity of Equisetin and Antibiotic Comparators Against MRSA

sample/disk label	concentration (% w/v)	inhibition zone (mm)
1	1	23.94 ± 0.66
2	0.5	21.75 ± 0.32
3	0.25	20.33 ± 0.73
4	0.125	14.74 ± 0.68
5	0.0625	10.94 ± 0.34
6	0.0313	9.11 ± 0.40
7	0.0156	7.33 ± 0.67
8	0.00781	7.03 ± 0.25
9	0.00391	–^a^
10	0.00195	–
Chloramphenicol (C)^b^	not applicable	24.71 ± 0.68
Amoxicillin (A)^c^	not applicable	11.76 ± 0.66
DMSO control (−)^d^	not applicable	–

a – = no inhibition; (*n* = 3).

b Positive control 1: Oxoid disks containing 30 μg chloramphenicol.

c Positive control 2: Oxoid disks containing 25 μg amoxicillin.

d Negative control: DMSO.

In the recorded disk-diffusion results, chloramphenicol (30 μg/disk) and amoxicillin (25 μg/disk) produced zones of 24.71 ± 0.68 and 11.76 ± 0.66 mm, respectively; DMSO produced no measurable zone. These antibiotic measurements are reported as descriptive reference values. Differences in disk loading and diffusion behavior prevent the zone diameters from establishing equivalent potency or superiority of Compound **1** over either antibiotic. The isolated compound was tested only against MRSA, so its antibacterial spectrum cannot be inferred from these results.

### Structural Characterization of Compound 1

In the spectroscopic characterization, the UV–vis spectrum (Figure 2) recorded in methanol showed a maximum at 291 nm (absorbance 0.510) and a minor peak at 220 nm (absorbance 0.440). Meanwhile, FTIR analysis (Figure 3) showed absorption bands associated with hydroxyl (3296.70 cm^–1^), aliphatic C–H (2917.52 cm^–1^), carbonyl (1646.88 cm^–1^), and conjugated C=C stretching (1505.91 cm^–1^) functional groups.

**Figure 2:**
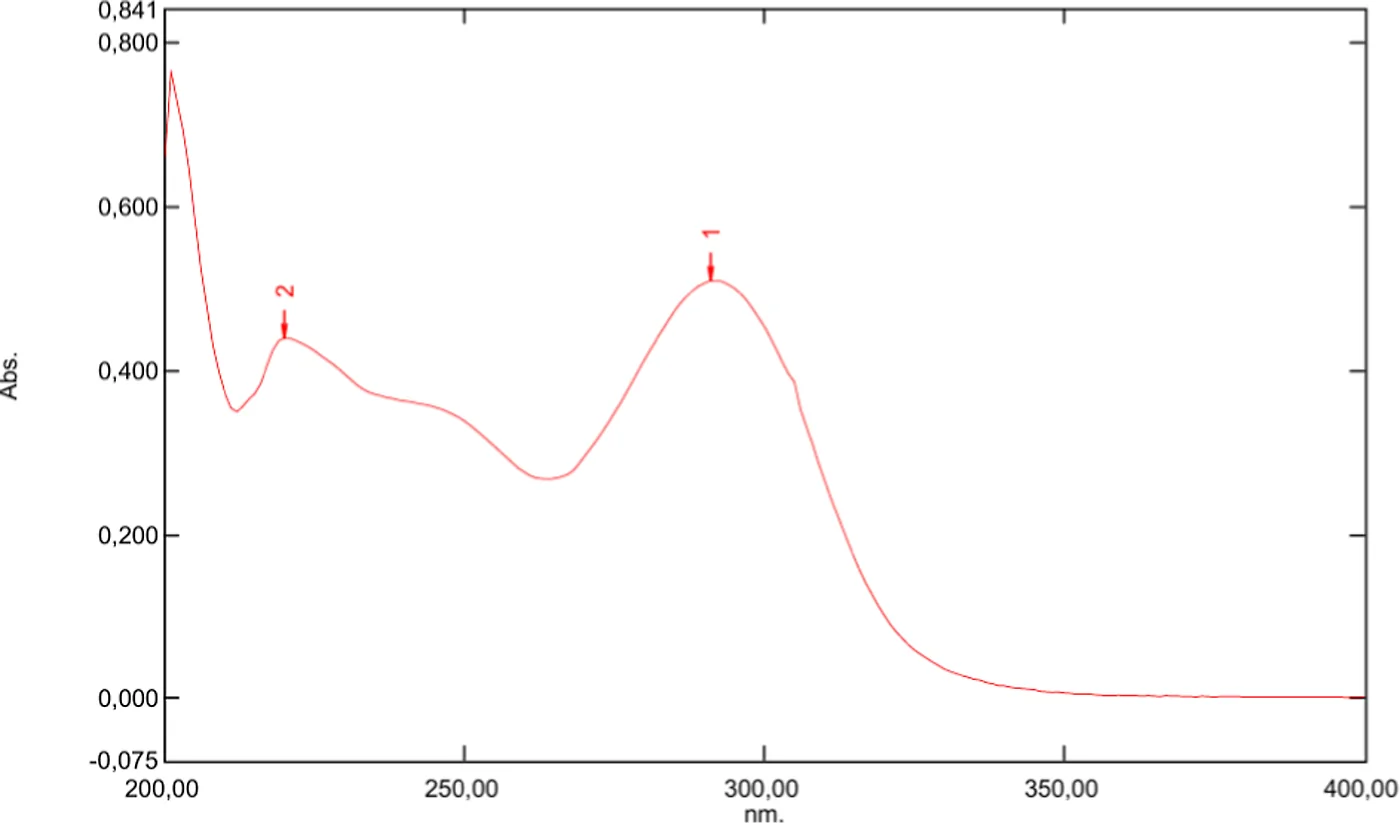
UV–vis spectrum of Compound **1** (equisetin), showing two absorption maxima: (1) at 291 nm (Abs = 0.510), and (2) at 220 nm (Abs = 0.440).

**Figure 3:**
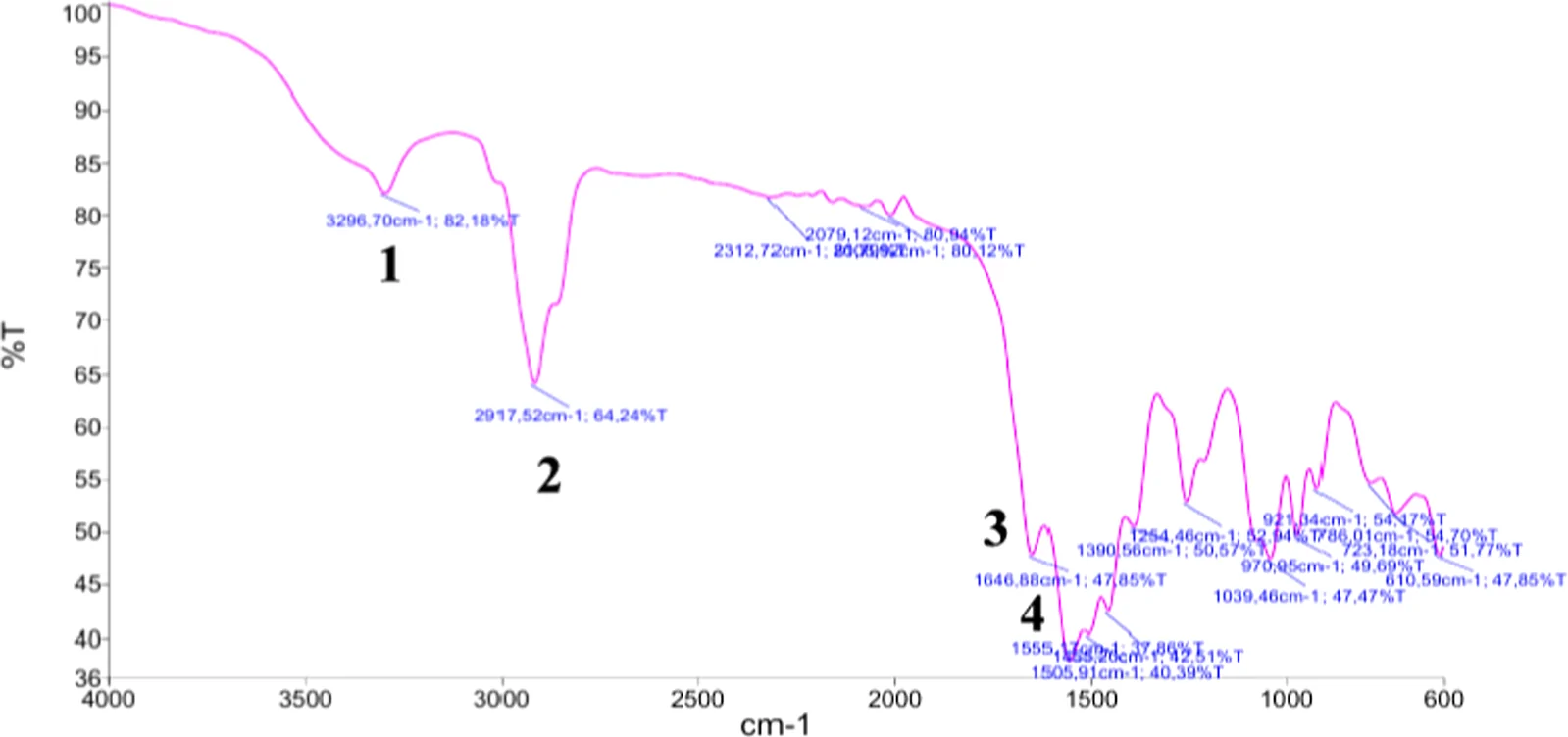
FTIR spectrum of Compound **1**, with bands assigned to (1) O–H, (2) aliphatic C–H, (3) C = O, and (4) conjugated C = C stretching.

LC–MS/MS analysis in positive-ion mode showed a chromatographic peak at 14.20 min and an ion at *m*/*z* 374.2343, assigned as [M + H]^+^ (calculated for C_22_H_32_NO_4_^+^, 374.2326). This assignment is consistent with the neutral formula C_22_H_31_NO_4_ (Figure 4). Product ions were observed at *m*/*z* 200.0934, 302.1767, 346.2389, and 356.2230.

**Figure 4:**
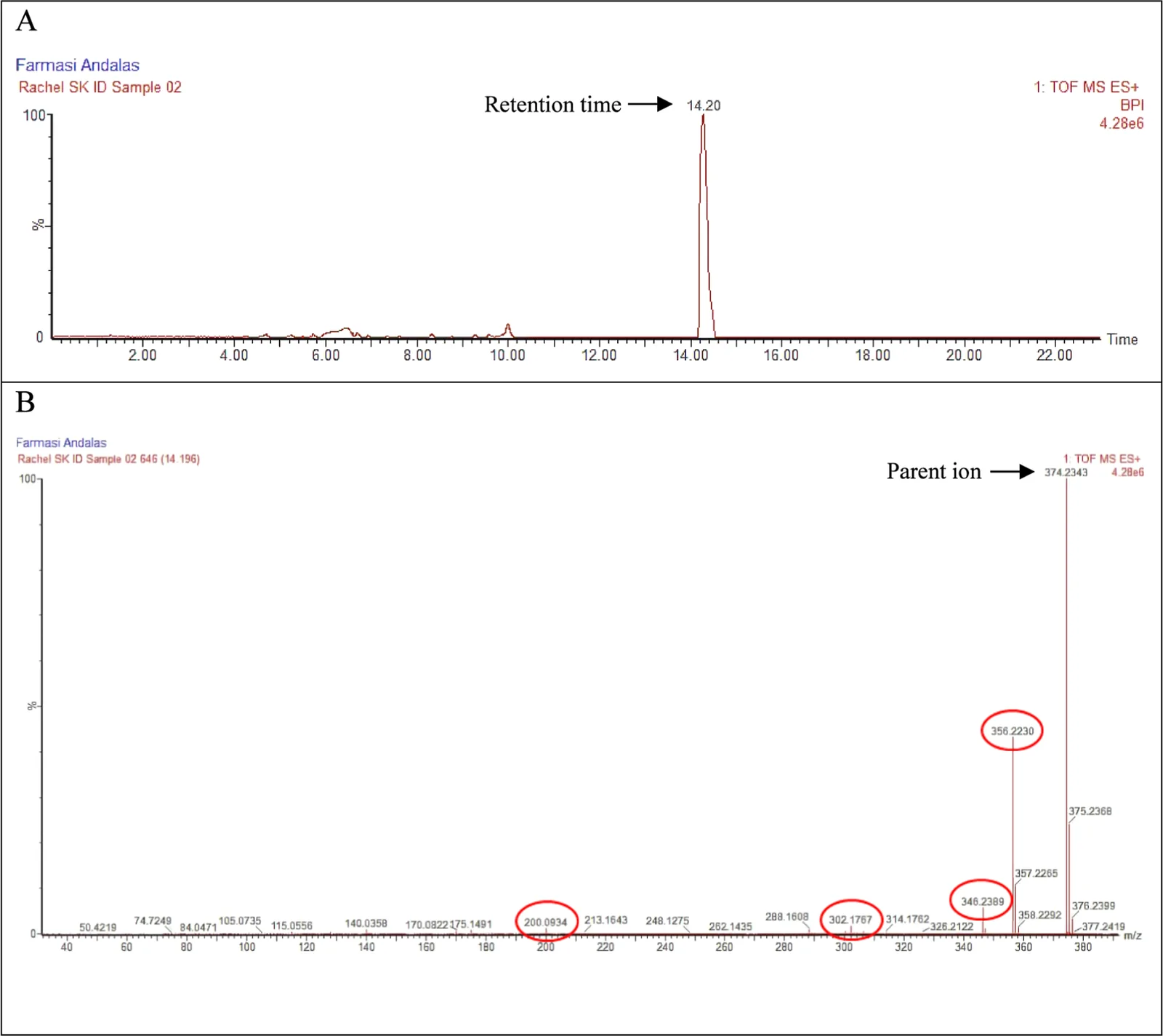
LC–MS/MS analysis of Compound **1**: (A) chromatogram showing a retention time at 14.20 min; (B) mass spectrum with a parent ion at *m*/*z* 374.2343 [M + H]^+^ and major fragment ions at *m*/*z* 200.0934, 302.1767, 346.2389, and 356.2230.

The ^1^H NMR spectrum (400 MHz, CD_3_OD; Table 2) contained four olefinic protons at δ_H_ 5.31, 5.35, and 5.15–5.29, an *N*-methyl singlet at δ_H_ 2.93, and methyl resonances at δ_H_ 1.29, 1.50, and 0.85. Signals at δ_H_ 3.90, 3.81, and 3.73 were assigned to the methine and hydroxymethyl protons of the tetramic acid moiety. The assigned resonances accounted for 29 nonexchangeable protons; exchangeable hydroxyl signals were not observed in CD_3_OD. Comparison with the equisetin data of Phillips et al.^14^ supported the assignment, although their spectra were acquired in CDCl_3_ at −20 °C, whereas the present spectrum was acquired in CD_3_OD at room temperature. Solvent, temperature, and tautomerism may contribute to the observed differences; these possibilities do not independently validate every assignment.

**Table 2:** Comparison of the ^1^H NMR Data of Compound **1** with Literature Data for Equisetin

position	compound **1**: δ_H_ (400 MHz, CD_3_OD)	literature equisetin: δ_H_ (500 MHz, CDCl_3_, −20 °C)^14^
3-H	3.35 (1H, m)	3.32 (1H, br d, *J* = 9.0 Hz)
4-H	5.31 (1H, br s)	5.40 (1H, br s)
5-H	5.35 (1H, br s)	5.40 (1H, br s)
6-H, 10a-H	2.13–2.19 (2H, m)	1.84 (1H, br t, *J* = 12.0 Hz); 1.96 (1H, br d, *J* = 12.0 Hz)
7a-H, 9a-H, 8-H, 11-H	1.60–1.66 (4H, m)	1.81 (1H, br d, *J* = 12.0 Hz); 1.76 (1H, br d, *J* = 12.0 Hz); 1.48 (1H, m); 1.66 (1H, br d, *J* = 11.0 Hz)
7b-H, 9b-H, 10b-H	1.07–1.11 (3H, m)	0.90 (1H, q, *J* = 12.0 Hz); 1.11 (1H, q, *J* = 12.0 Hz); 1.04 (1H, q, *J* = 12.0 Hz)
12-CH_3_	1.29 (3H, s)	1.46 (3H, s)
13-H, 14-H	5.15–5.29 (2H, m)	5.17 (1H, dd, *J* = 15.0, 9.0 Hz); 5.25 (1H, dq, *J* = 15.0, 6.5 Hz)
15-CH_3_	1.50 (3H, d, *J* = 6.0)	1.56 (3H, d, *J* = 6.5 Hz)
16-CH_3_	0.85 (3H, d, *J* = 6.4)	0.92 (3H, d, *J* = 6.5 Hz)
5′-H	3.90 (1H, m)	3.67 (1H, dd, *J* = 5.0, 3.5 Hz)
6′a-H	3.81 (1H, dd, *J* = 11.6, 4.0)	4.07 (1H, dd, *J* = 12.0, 3.5 Hz)
6′b-H	3.73 (1H, dd, *J* = 11.6, 4.8)	3.90 (1H, dd, *J* = 12.0, 5.0 Hz)
7′-NCH_3_	2.93 (3H, s)	3.07 (3H, s)

The UV–vis, FTIR, LC–MS/MS, and ^1^H NMR data (Table 3) support assignment of Compound **1** as equisetin by comparison with published data.^15,16^ The proposed structure is shown in Figure 5. Because ^13^C and two-dimensional NMR data were not acquired, the full connectivity and stereochemical assignment were not independently established in this study.

**Table 3:** Summary of the Characterization Data of Compound **1** (Equisetin)

characterization	analytical result
organoleptic	white powder
TLC profile	EtOAc/DCM (4:1) + 3 drops formic acid, *R*_f_ = 0.9
	toluene/acetone (3:1), *R*_f_ = 0.6
	toluene/DCM (1.5:3.5), *R*_f_ = 0.4
UV–vis spectrophotometer (nm)	λ_max_ (in MeOH) at 291 nm
FTIR wavenumber (cm^–1^)	3296.70 (−OH); 2917.52 (aliphatic C–H); 1646.88 (C=O); 1505.91 (conjugated C=C stretching)
parent ion (*m*/*z*) [M + H]^+^	374.2343
fragment ions (*m*/*z*)	200.0934; 302.1767; 346.2389; 356.2230

**Figure 5:**
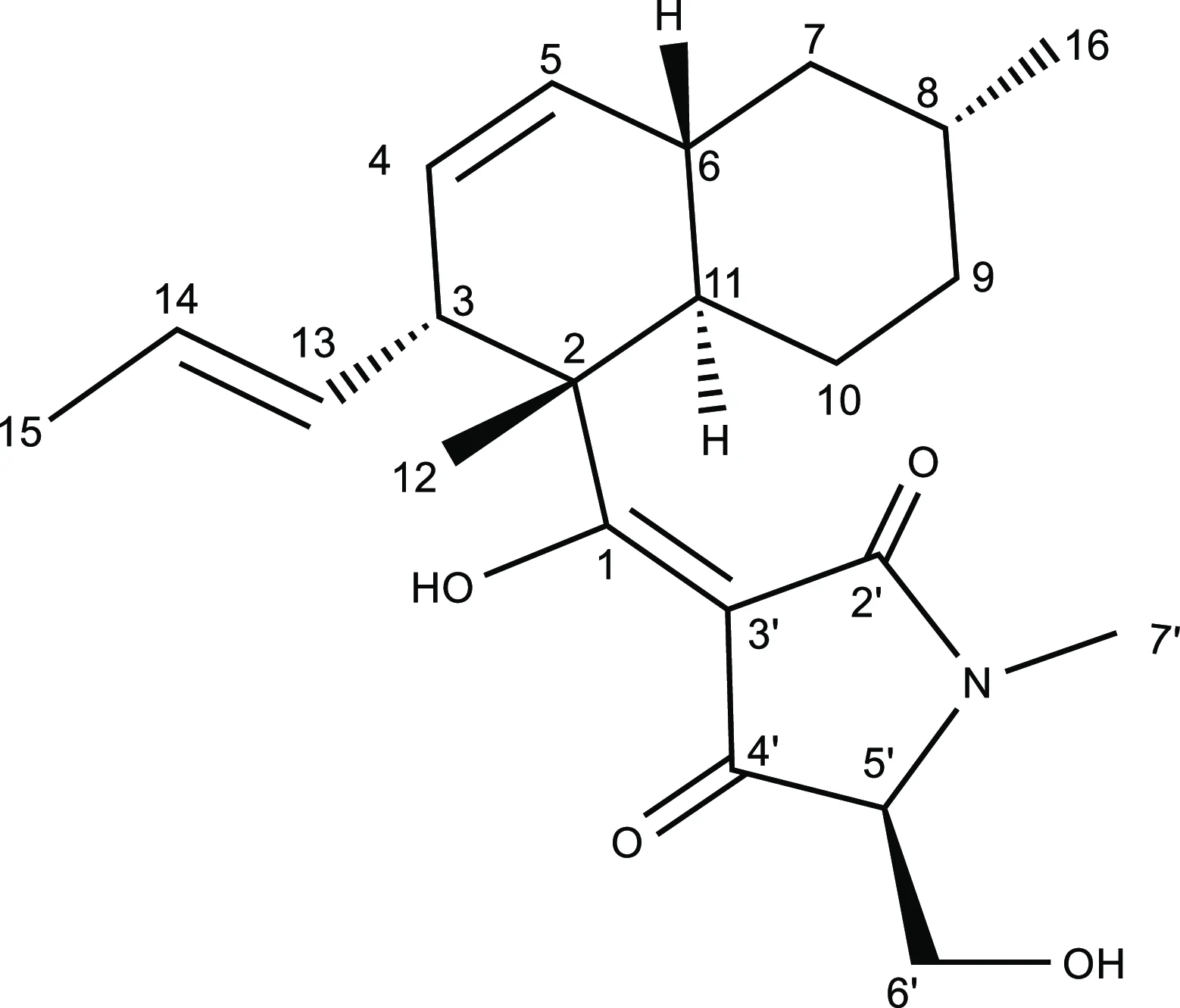
Proposed equisetin structure (C_22_H_31_NO_4_) used for comparison with the isolated compound. Stereochemistry was not independently established in this study.

Equisetin is a tetramic acid metabolite originally reported from *Fusarium equiseti*.^17^ Previous work has described antibacterial activity, including activity against resistant bacteria,^18^ and host-mediated effects against intracellular *S. aureus*.^19^ Those findings provide biological context but do not establish the mechanism operating in the present disk-diffusion assay.

Previous equisetin studies reported MICs against MRSA.^15,20^ The present value of 78.1 μg/mL is the lowest tested solution concentration with a measurable disk-diffusion zone and cannot be directly compared with dilution-based MICs. The reported data set contains neither an MIC for Compound **1** nor comparative MICs for chloramphenicol and amoxicillin. Standardized MIC measurements are needed to quantify antibacterial potency relative to the antibiotics. The current data support anti-MRSA activity under the tested conditions, without establishing clinical susceptibility, antibacterial spectrum, or therapeutic efficacy.

### Molecular Docking Analysis

Piperacillin and equisetin yielded docking scores of −6.1242 and −5.5041 kcal/mol, respectively (Table 4). These are model-dependent scores and are not experimentally measured binding free energies.

**Table 4:** Molecular Docking Results

compound	S score (kcal/mol)	RMSD (Å)	hydrogen bonding	van der Waals interaction	C–H bond	amino acid residue (hydrogen bond)
piperacillin (reference)	–6.1242	3.1282	5	9	0	Ser403, Lys406, Asn464, Gln521
equisetin	–5.5041	1.6339	2	12	0	Glu460, Ser461

The piperacillin pose was located in the catalytic region of PBP2a and contained five predicted hydrogen bonds involving Ser403, Lys406, Asn464, and Gln521 (Figure 6). Other predicted contacts included van der Waals, electrostatic, and hydrophobic interactions. This provides a structural reference for the modeled equisetin pose; docking alone does not establish enzyme inhibition or antibacterial efficacy.

**Figure 6:**
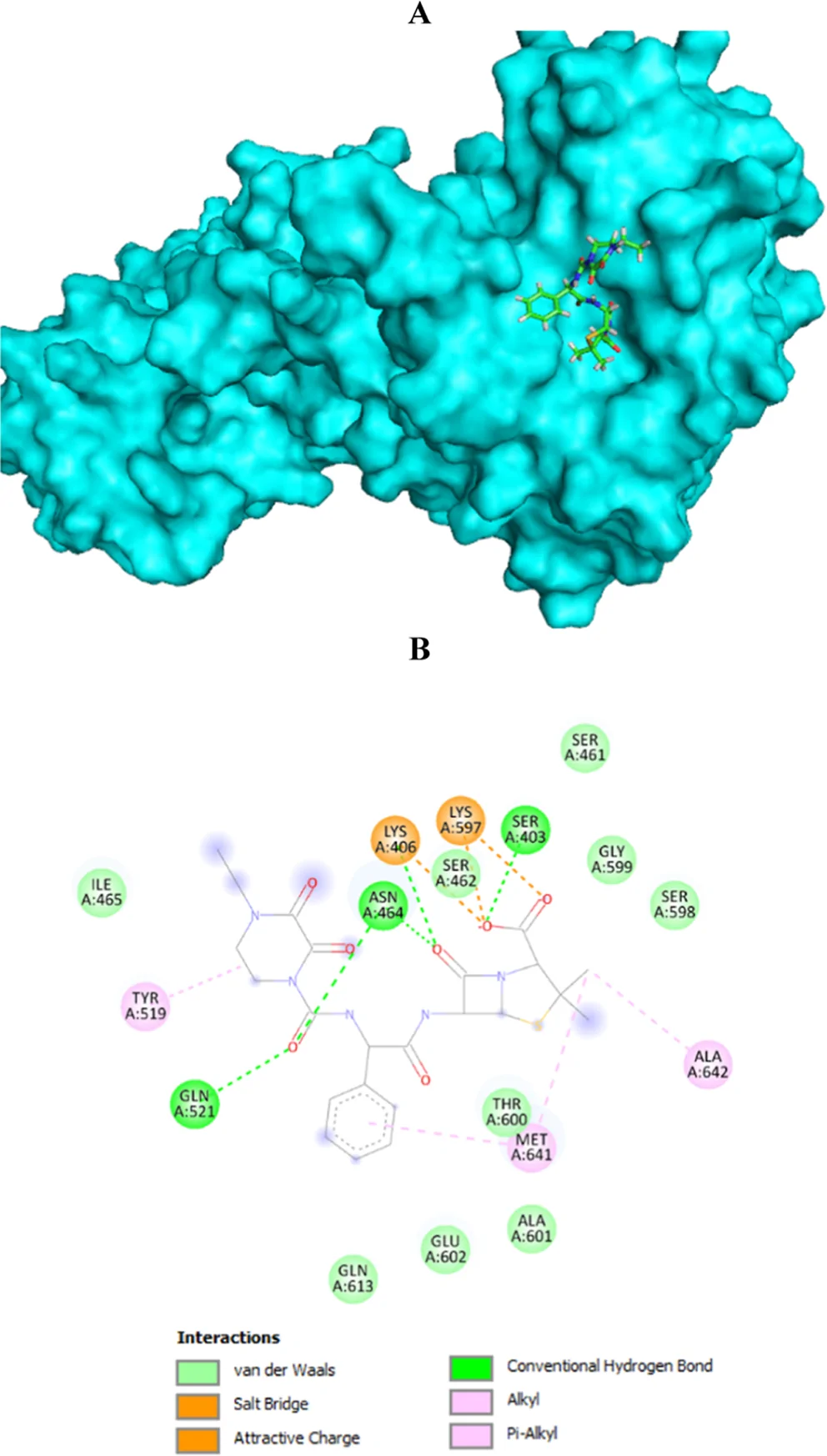
Docking interaction of piperacillin with PBP2a (PDB ID: 6H5O). (A) Three-dimensional surface representation of PBP2a showing piperacillin bound in the canonical binding pocket. (B) Two-dimensional interaction diagram depicting hydrogen bonding, salt bridge, and hydrophobic interactions of piperacillin with key residues, including Ser403 and Asn464.

The reported equisetin pose was outside the canonical catalytic pocket (Figure 7), with two predicted hydrogen bonds to Glu460 and Ser461 and other contacts involving Met641, Ala642, and His583. This pose does not establish binding at a functional allosteric site.

**Figure 7:**
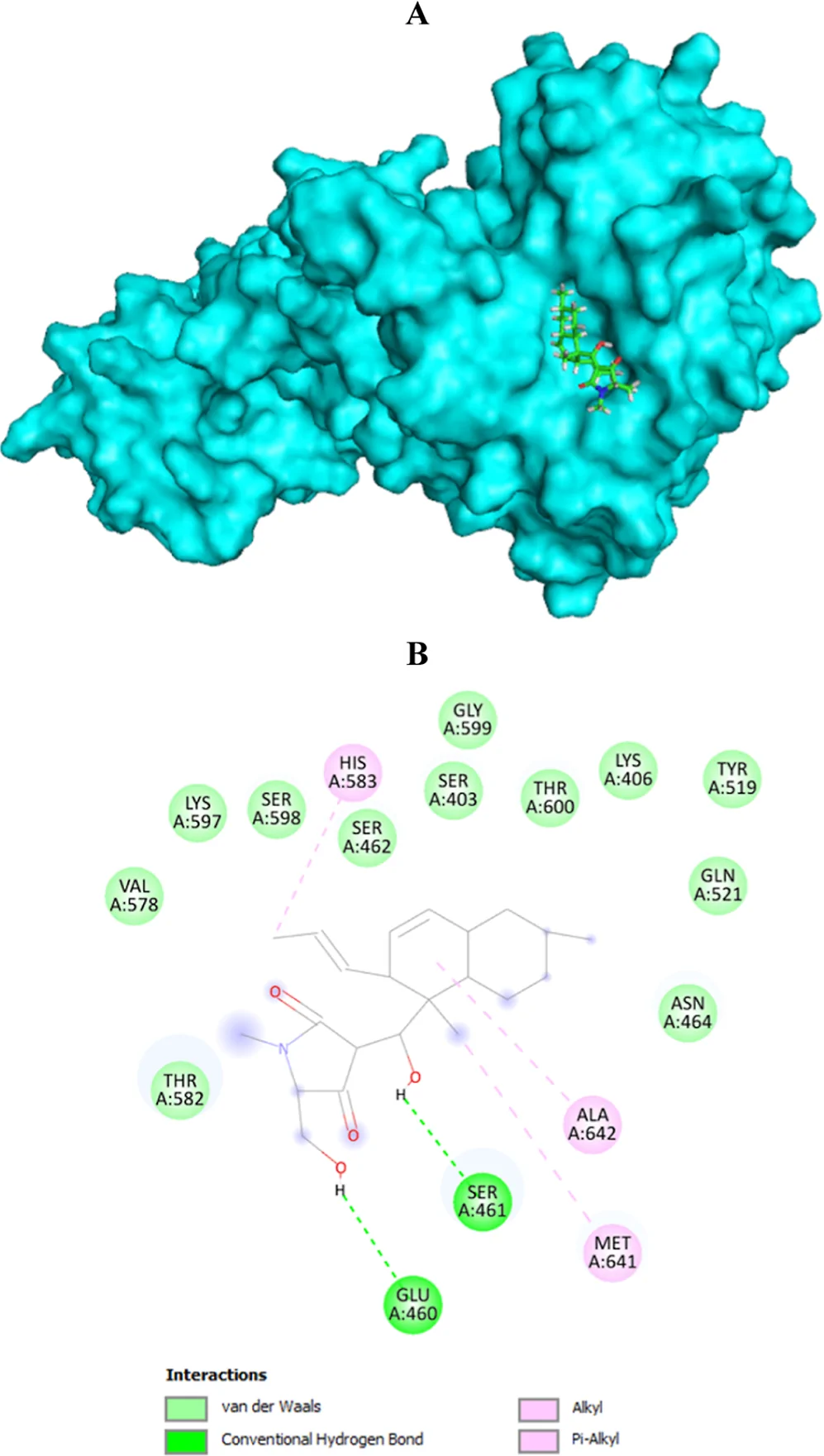
Docking interaction of equisetin with PBP2a (PDB ID: 6H5O). (A) Three-dimensional surface representation of PBP2a showing equisetin bound outside the canonical active site pocket. (B) Two-dimensional interaction diagram depicting hydrogen bonding and hydrophobic interactions of equisetin with surrounding residues, including Glu460, Ser461, Met641, Ala642, and His583.

The docking results are hypothesis-generating. The antibacterial assay does not identify PBP2a as the functional target of Compound **1**, and direct binding and functional evidence would be needed to link the predicted interaction to anti-MRSA activity.

### Molecular Dynamics Analysis

Over the 100 ns trajectories, the equisetin and reference complexes showed broadly comparable protein backbone RMSD profiles (Figure 8A). The equisetin-complex backbone RMSD was approximately 0.18–0.28 nm for much of the trajectory. This describes protein conformational variation and does not by itself demonstrate that the ligand remained in a particular binding pose.

**Figure 8:**
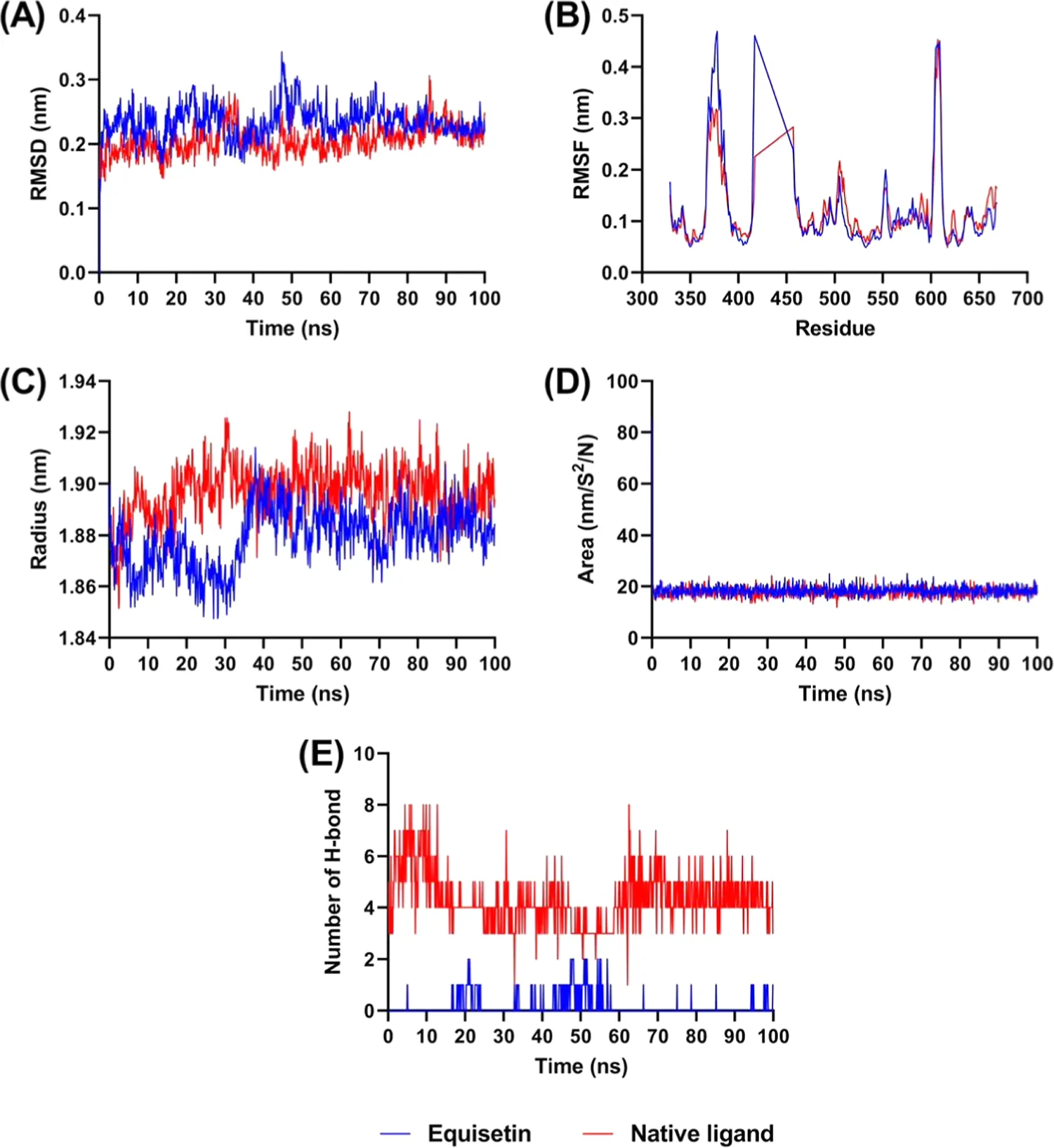
Molecular dynamics analysis over 100 ns for the equisetin and reference protein complexes: (A) protein backbone RMSD; (B) residue RMSF; (C) radius of gyration; (D) solvent-accessible surface area; and (E) protein–ligand hydrogen-bond counts. The reference trace is labeled “Native ligand” in the supplied plot.

The RMSF profiles showed similar patterns of residue flexibility, with larger fluctuations in selected regions (Figure 8B). Rg and SASA profiles described changes in protein compactness and surface exposure during the trajectories (Figure 8C,D). Equisetin generally formed 0–2 protein–ligand hydrogen bonds, whereas the reference ligand generally formed 4–8 (Figure 8E). Hydrogen-bond counts alone do not quantify binding affinity or establish which interactions dominate binding.

These simulations describe the behavior of the modeled complexes within the sampled trajectories. They do not demonstrate persistent ligand residence, an allosteric mechanism, functional inhibition of PBP2a, or a causal relationship with the observed anti-MRSA activity. Such conclusions require additional analyses and experimental evidence.

The principal limitations are the absence of standardized MIC measurements, evaluation of only one reported MRSA isolate, incomplete independent structural confirmation, and the exploratory nature of the computational analysis. These limitations should guide subsequent validation of the isolated compound.

## Conclusion

Compound **1** was isolated from *F. incarnatum* JMB4 associated with *J. multifida* and assigned as equisetin on the basis of the available spectroscopic data. The compound produced measurable inhibition zones against MRSA, including 23.94 ± 0.66 mm at 1% (w/v). The lowest tested solution concentration with a measurable zone was 78.1 μg/mL; this diffusion end point is not an MIC. Chloramphenicol and amoxicillin provided reference zone measurements, but unequal disk loads and the absence of comparative MICs prevent conclusions about relative antibacterial potency. Docking and molecular dynamics provide exploratory descriptions of a possible PBP2a interaction without establishing target inhibition. Further structural confirmation and standardized antibacterial evaluation are required.

## Data Availability

The data supporting the findings of this study are available within the article.

## Abbreviations

FTIR: Fourier-transform infrared spectroscopy
UV–vis: ultraviolet–visible spectroscopy
LC-MS/MS: liquid chromatography–tandem mass spectrometry
MRSA: methicillin-resistant *Staphylococcus aureus*
MIC: minimum inhibitory concentration
DMSO: dimethyl sulfoxide
DCM: dichloromethane
MeOH: methanol
EtOAc: ethyl acetate
